# Psychiatric partner resemblance and its socioeconomic component across the life course

**DOI:** 10.64898/2026.09.11.26362836

**Published:** 2026-09-13

**Authors:** Saeid Rasekhi Dehkordi, Martin Dalgaard Villumsen, Dorte Helenius, Wouter Peyrot, Thomas Werge, Alfonso Buil, Abdel Abdellaoui

## Abstract

Partner resemblance influences and reflects the social, genetic and health structure of human populations. Using Danish registers on 787,658 couples, we quantified partner resemblance for ten psychiatric disorders and socioeconomic status (SES), measured up to a year before first childbirth and across the life course. Psychiatric partner correlations ranged from 0.08 for eating disorders to 0.36 for schizophrenia, below those for education (0.63) and income (0.57). Income resemblance fell substantially across the life course (to 0.38), whereas education resemblance was stable. Psychiatric resemblance changed little across windows. Adjusting psychiatric liability for own SES reduced psychiatric partner resemblance, whereas adjusting SES for own psychiatric status barely affected SES partner resemblance. The reduction in psychiatric partner resemblance had a median of ∼7% before first childbirth but ∼26% across the life course, as diagnosis and SES became increasingly coupled within individuals. What psychiatric partner resemblance represents therefore depends on when it was measured.

## Full Text Availability

The license terms selected by the author(s) for this preprint version do not permit archiving in PMC. The full text is available from the preprint server.

